# Maternal and Postnatal Effects of Poly I:C‐Induced Intrauterine Inflammation and Rytvela, a Non‐Competitive IL‐1R Antagonist, in the Pregnant Spiny Mouse

**DOI:** 10.1002/dneu.70064

**Published:** 2026-09-17

**Authors:** Nhi T. Tran, Rachid Kamareddine, Nadia Bellofiore, David M. Olson, Sylvain Chemtob, Sarah A. Robertson, Jeffrey A. Keelan, Stacey J. Ellery

**Affiliations:** ^1^ The Ritchie Centre, Department of Obstetrics and Gynaecology Monash University Clayton Victoria Australia; ^2^ Hudson Institute of Medical Research Clayton Victoria Australia; ^3^ Departments of Obstetrics and Gynecology, Pediatrics, and Physiology University of Alberta Edmonton Alberta Canada; ^4^ Department of Pharmacology and Physiology Université de Montréal, Research Center, CHU Sainte‐Justine Montreal Quebec Canada; ^5^ School of Pharmacy and Biomedical Science Adelaide University Adelaide South Australia Australia; ^6^ School of Biomedical Sciences University of Western Australia Perth Australia

**Keywords:** behavior, cognitive function, cytokines, maternal immune challenge, offspring growth, pregnancy

## Abstract

Maternal infection‐induced inflammation during pregnancy is associated with adverse neurodevelopment. Rytvela, an allosteric IL‐1 receptor antagonist, reduces IL‐1β‐mediated pathology in fetal rodents and sheep. However, its effects on TLR3‐mediated inflammation and subsequent offspring growth and behavior remain unclear. Using precocial spiny mice, the primary outcome of this study was to examine whether rytvela could attenuate the acute maternal inflammatory response to mid‐gestation exposure to polyinosinic:polycytidylic acid (Poly(I:C)). Secondary offspring outcomes included postnatal growth and behavior. At gestational day 20 (term = 39 days), pregnant mice received two intraperitoneal injections, 4 h apart, of saline (0.9% w/v, *n* = 14), Poly(I:C) (12 mg/kg, *n* = 14), or Poly(I:C) + rytvela (12 mg/kg + 3 mg/kg, respectively, *n* = 14). Ten dams/group were culled 8 h after dose 1 to assess inflammatory responses. The remainder (*n* = 4 dams/group) were delivered at term. Offspring (control *n* = 7, Poly(I:C) *n* = 8, Poly(I:C) + rytvela *n* = 11) growth was monitored and behavioral testing undertaken from postnatal day (PND) 3–45. Poly(I:C) increased maternal serum (*p* = 0.01) and amniotic fluid (*p* = 0.003) IL‐1β, and upregulated *Il1b*, *Il6* and *Tnf* expression in the spleen and thymus. Rytvela reduced IL‐1β levels but did not attenuate elevated cytokine gene expression. Poly(I:C) slowed postnatal growth (PND 18–27, *p* < 0.05), which recovered earlier with rytvela. Poly(I:C) exposure did not alter offspring behavior in tests completed to PND 45. However, Poly(I:C) + rytvela offspring showed reduced locomotor activity (distance: *p* < 0.001; velocity: *p* = 0.021). In conclusion, rytvela attenuated the primary inflammatory response to Poly(I:C), promoting earlier postnatal growth recovery despite persistent cytokine gene expression. Reduced locomotor activity after prenatal rytvela exposure warrants further investigation.

## 1 Introduction

Infection‐induced inflammation during pregnancy significantly contributes to neonatal mortality, hospitalization, and neurodevelopmental disorders (Liu et al. 2012). Epidemiological studies correlate maternal intrauterine infection with an increased risk of postnatal neurocognitive deficits (Brown 2006; Brown, Cohen et al. 2000; Brown et al. 2000; Brown et al., 2004, 2005), and experimental models link pro‐inflammatory agents from the maternal immune response following infection to altered offspring development and physiological function (Ratnayake et al. 2013).

Infection and inflammation involve the production of pro‐inflammatory cytokines, notably interleukin‐1β (IL‐1β), which is recognized as a primary driver of the inflammatory cascade (de Jong et al. 2002). Most nucleated cells in the vicinity of a microbial stimulus can secrete IL‐1β. In particular, macrophages and dendritic cells produce IL‐1β to stimulate the production of other immunomodulatory factors from neighboring cells and at distal sites (Chung et al. 2009). This is evident in murine studies, where even modest doses of immune stimulants, such as the viral mimetic and toll‐like receptor‐3 (TLR3) agonist polyinosinic:polycytidylic acid [Poly(I:C)], lead to increased circulating IL‐1β levels and impaired memory and social skills in offspring (Arrode‐Brusés and Brusés 2012; Potter et al. 2025; Luo et al. 2026). Clinical studies report increased IL‐1β in placental tissues (Taniguchi et al. 1991) and maternal serum (Romero et al. 2016) in cases of infection and inflammation during human pregnancy. Consequently, IL‐1β is considered a key target for therapeutic intervention to improve neurodevelopmental outcomes in pregnancies complicated by maternal infection (Kelly et al. 2023).

Rytvela is a 7‐amino acid peptide that acts as a non‐competitive antagonist of IL‐1 receptor (IL‐1R) activation (Quiniou, Sapieha et al. 2008; Nadeau‐Vallée et al. 2015). It exerts allosteric effects on IL‐1β signaling, inhibiting p38 MAPK and ROCK2 signaling while preserving the NF‐κB pathway (Nadeau‐Vallée et al. 2015). Murine models of preterm birth have shown rytvela to be an efficient antenatal intervention for reducing inflammation and averting untimely fetal membrane rupture following direct exposure to IL‐1β or various microbial agents that activate TLR2/TLR4 signaling pathways (Quiniou et al. 2008; Nadeau‐Vallée et al. 2017; Chin et al. 2025). In a sheep model of intraamniotic inflammation caused by in utero exposure to *Escherichia coli* lipopolysaccharides (LPS) and TLR4 signaling, rytvela reduced cytokine and chemokine levels in the chorioamniotic membranes and partially inhibited histological brain injury in the preterm fetus (Takahashi et al. 2021, 2025).

Rytvela has a favorable pharmacological profile, with no evidence of acute biological or reproductive toxicity (Nadeau‐Vallée et al. 2017). Studies in mice suggest that it may have better specificity, stability, and efficacy than currently approved IL‐1 antagonists (Nadeau‐Vallée et al. 2015, 2017). The greater selectivity of rytvela decreases the risk of off‐target and adverse events, while its improved bioavailability may enable administration via less invasive routes (Quiniou et al. 2008).

The impact of maternal rytvela administration following prenatal exposure to inflammation driven by a maternal TLR3‐mediated response has not yet been assessed. Therefore, the primary aim of this study was to evaluate the ability of rytvela administration to reduce the maternal inflammatory cascade and, secondarily protect against negative postnatal neurodevelopmental sequelae. Specifically, we used the spiny mouse model (*Acomys cahirinus*) of prenatal infection and inflammation induced by mid‐gestation maternal exposure to the viral mimetic and TLR3 agonist Poly(I:C). This maternal immune challenge results in neuroinflammation and abnormal postnatal behavior in offspring (Ratnayake et al. 2014). A low dose of Poly(I:C) was selected to mimic a modest maternal viral infection, given the common and increasing risk of exposure to viruses such as SARS‐CoV‐2 and influenza in pregnant women (Yu et al. 2022). Unlike other murine models, the spiny mouse has a longer gestation (38–39 days), producing precocial offspring capable of feeding, locomotion, sight, and smell at birth (Brunjes 1989, 1990). Hence, behavioral and developmental assessments on spiny mouse offspring can be undertaken at early postnatal ages without maternal rejection and have previously been used to successfully model human newborn neurodevelopmental outcomes (Ratnayake et al. 2014). We hypothesized that antenatal administration of rytvela would reduce IL‐1β‐induced maternal inflammation at the time of the insult and consequently improve postnatal growth and behavior.

## 2 Materials and Methods

### 2.1 Animal Experiments

All experiments were approved in advance by the Monash University Animal Ethics Committee (MMCA/2020/14) and conducted in accordance with the Australian Code of Practice for the Care and Use of Animals for Scientific Purposes. The experiments are reported in accordance with the ARRIVE (Animal Research: Reporting of In Vivo Experiments) guidelines for animal research. Spiny mice (*A. cahirinus*) from an in‐house colony established at Monash Medical Centre (Australia) were bred, housed, and time‐mated in a temperature‐controlled room (25±1°C, humidity 40%–50%) under a 12‐h light/dark cycle (0700 and 1900).

### 2.2 Treatment Administration

**FIGURE 1:**
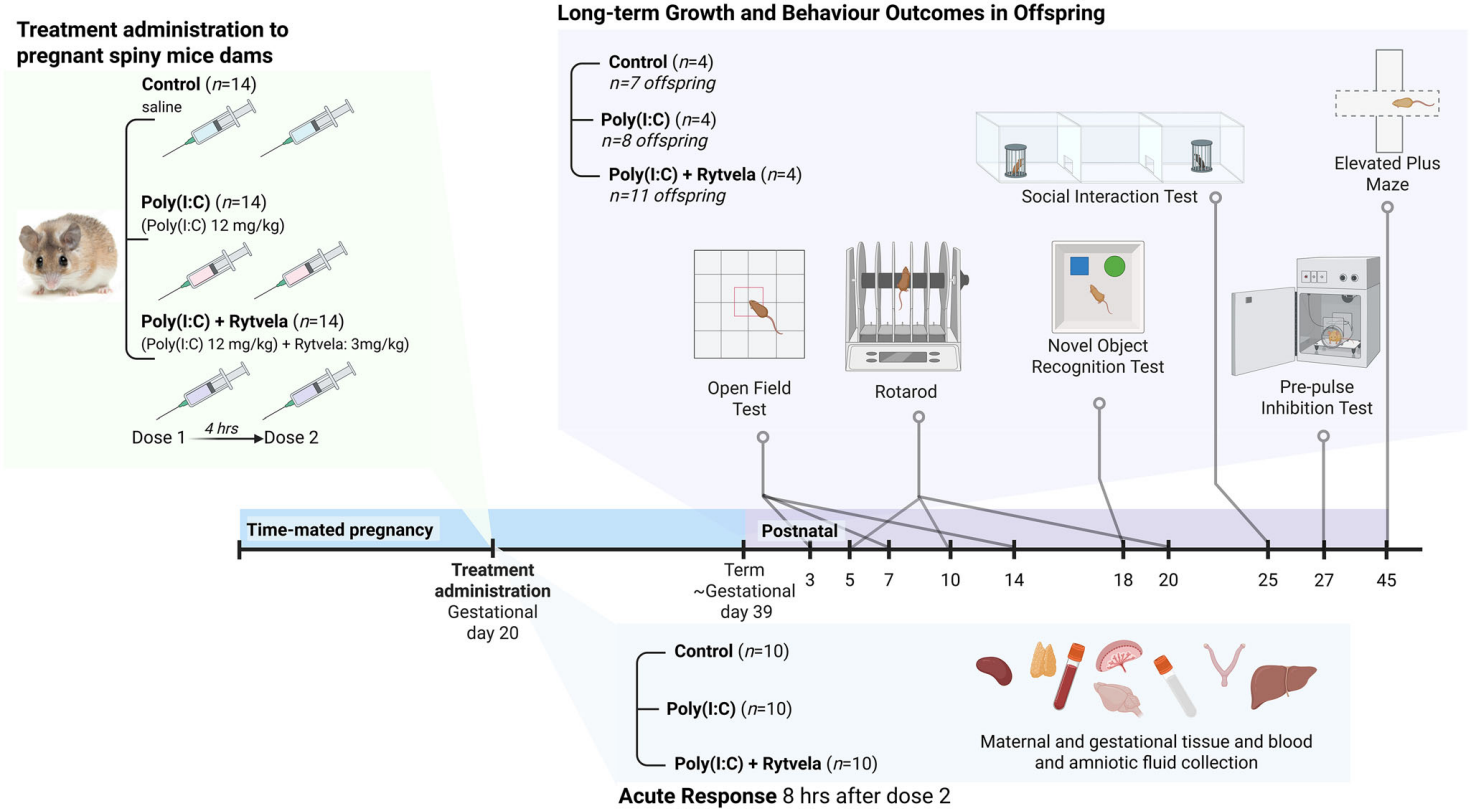
Schematic overview of experimental design. Pregnant spiny mouse dams at mid‐gestation received two doses of Poly(I:C), Poly(I:C) + rytvela, or saline (control) 4 h apart (*n* = 14/treatment). At 8 h after the first treatment, 10 dams/treatment were culled to assess acute maternal and fetal responses. The remaining dams (*n* = 4/treatment) were left to litter spontaneously at term. Offspring then underwent a series of behavior testing from postnatal day 3 to postnatal day 45.

A schematic overview of the experimental procedure is presented in Figure 1. At mid‐gestation (day 20 of ∼39‐day gestational length), pregnancy was confirmed in established breeders based on the date of their previous litter, body weight, and abdominal palpation (note spiny mice do not produce a copulatory plug). Dams were then randomly allocated to one of three groups:

1. Poly(I:C): 2 × doses of Poly(I:C) (12 mg/kg; Sigma, #P1530) dissolved in ∼150 µL of saline (0.9% NaCl, pH 7.4) and delivered 4 h apart, *n* = 14
2. Poly(I:C) + rytvela: 2 × doses of Poly(I:C) (12 mg/kg) and rytvela (3 mg/kg; Elim Biopharmaceuticals, CA, USA) dissolved in ∼150 µL of saline and delivered 4 h apart, *n* = 14
3. Control: 2 × doses of isovolumetric saline delivered 4 hours apart, *n* = 14

For all groups, the first treatment dose was administered at 0900 hours, with a second dose administered at 1300 hours. Two doses of Poly(I:C) were used as pilot testing revealed that two doses were required to produce reproducible and measurable maternal inflammatory responses. All dams were restrained in the supine position before intraperitoneal injections using a 25G needle. Body temperature was measured before and 2 h after the first intraperitoneal injection via a rectal probe. Dams were monitored for any febrile response, abnormal behavior (i.e., reduced alertness), and/or vaginal bleeding until post‐mortem.

### 2.3 Post‐Mortem Weights and Tissue Collection for Acute Response Analyses

At 8 h after the first treatment (i.e., 1700 hours), a post‐mortem was undertaken, whereby pregnant dams (*n* = 10/group) were euthanized via isoflurane overdose, and blood was collected via cardiac puncture. Blood was allowed to clot at room temperature for 30 min and then centrifuged at 2200 *g* for 10 min at 4°C to separate the serum. The maternal spleen and thymus were excised, weighed, and whole tissues snap‐frozen on dry ice; the maternal brain and liver were also weighed. The uterus was removed, and the number and location of fetuses and any fetal resorptions or overt abnormalities were recorded. Amniotic fluid was collected, and then individual fetal and placental units were excised and fetal body and placenta weight recorded and reported as placenta:fetal ratio. Placentae were dissected into the labyrinth and junctional zones before freezing. Serum, amniotic fluid, and tissues were stored at −80°C until analysis.

### 2.4 Gene Expression Analysis

Gestational tissues (uterus and placentae) and key maternal immune organs (spleen and thymus) were homogenized in RNA extraction buffer using reagents supplied in PureLink RNA mini kits (ThermoFisher Scientific #12183018A), according to the manufacturers’ instructions. Total RNA was extracted and DNase‐treated using on‐column PureLink DNase (ThermoFisher Scientific #12185010). cDNA was produced using the Invitrogen SuperScript III First‐Strand Synthesis System (ThermoFisher Scientific #18080051). Relative mRNA expression was analyzed for gene expression of cytokines *Il1b, Il6*, and *Tnf* using SYBR green PCR master mix (Applied Biosystems #4309155), normalized to the housekeeping gene 18S ribosomal RNA 1 *(Rn18s1)* (see Table S1 for primer sequences). For qPCR analysis, samples were run in duplicate. Denaturation occurred at 95°C for 10 min, followed by 40 amplification cycles of 95°C for 15 s and 60°C for 1 min for primer annealing and extension. Levels of mRNA expression relative to the housekeeping gene (*Rn18s1*) were determined for each sample using the 2^−ΔΔCT^ method. Gene expression data are reported as median and interquartile range (IQR).

### 2.5 IL‐1β Protein Analysis

Concentrations of IL‐1β in maternal serum and amniotic fluid samples were measured using a commercially available mouse ELISA kit (RayBiotech, #ELM‐IL1β). The kit was used according to the manufacturer's instructions and validated for the spiny mouse using spike and recovery tests (Tables S2 and S3). Due to inadequate sample collection, one control maternal serum and amniotic fluid sample and three Poly(I:C) amniotic fluid samples could not be analyzed.

### 2.6 Long‐Term Growth and Behavior Outcomes in Offspring

A subset of control, Poly(I:C), and Poly(I:C) + rytvela dams (*n* = 4/treatment) were randomly selected to litter naturally at term (39 days gestational age) (see Figure 1). Random allocation of dams to treatments and different litter sizes resulted in seven pups in the saline‐control group, eight pups in the Poly(I:C) group, and 11 pups in the Poly(I:C) + rytvela group. These pups were subjected to a series of validated behavioral assessments at various time points until postnatal day (PND) 45. All tests were carried out between 0800 and 1200 hours. The designated PND of the test was always within ±2 days of the specified age. Behavioral testing was conducted in a room separate from the housing room. The body weight of each pup was recorded before testing. Testing arenas were thoroughly cleaned with 70% ethanol between tests. All testing was conducted under lighting levels set at 2.8 lux. Animals were tested in a randomized order by an experimenter blinded to the group allocation. All mouse activity was recorded using an overhead HD digital CCTV video camera and continuously tracked with the CleverSys TopScan software (Reston, VA, USA). All post‐test behavior analyses of activity were conducted using an automated and unbiased CleverSys TopScan software.

#### 2.6.1 Open Field Test

Open field tests (OFTs) were conducted on PND 3, 7, and 14 to assess locomotor, exploratory, and anxiety‐like behaviors, as previously described (Ratnayake et al. 2014; Tran et al. 2025). Briefly, spiny mice were placed in the center of a 40 × 40 cm black open‐field apparatus with 50‐cm high walls and allowed to explore freely for 10 min. The open‐field apparatus was divided into a central zone, defined as the inner zone (20 × 20 cm), and a peripheral zone, defined as the outer zone. Movement was tracked throughout the open‐field trial by identifying nose, body, and tail movements using an overhead camera located above the apparatus. Thigmotaxis, or “wall hugging” behavior, was measured as total duration (seconds) in the outer zone. Total duration (seconds) in the inner zone was also recorded. The total distance travelled (mm) and average velocity of movement (mm/s) in the open‐field apparatus were measured to assess general locomotor activity.

#### 2.6.2 Rotarod

Rotarod tests were conducted on PND 5, 10, and 20 to assess motor skills, memory, and endurance, as previously described (Ratnayake et al. 2014). A standard accelerating Rotarod apparatus was modified by adding 12 plastic rungs, placed 1.5 cm above the surface of the inner axle, allowing the mouse to correctly place all four paws on the rungs to stay on the apparatus as it rotated. The mouse was placed on the rungs when the apparatus was at rest, and the speed was then increased by 7.2 revolutions/min. All animals were allowed two learning (or habituation) runs on the rotarod, and then the latency to fall was determined over five trials. After trials 1 and 2 were completed, the animals were allowed a 10‐min rest period before commencing trials 3–5; this rest interval was interposed to test the ability of the animal to retain or improve on the level of performance reached after T2. The latency to fall off the rotarod (s) was recorded for each trial; a maximum latency of 5 min was allowed for each trial.

#### 2.6.3 Novel Object Recognition Test

Novel object recognition test (NORT) was conducted on PND 18 to assess non‐spatial memory and learning behavior and novel object exploration (Lueptow 2017), as previously described (Ratnayake et al. 2012, 2014; Tran et al. 2025). Briefly, mice were placed in the same open‐field arena (40 × 40 × 50 cm) and allowed to explore freely for 10 min, acting as a habituation trial. Two plastic children's blocks of identical shape, color, and size were then placed in the arena, and mice were allowed to freely explore for 10 min in session 1: familiarization (learning) period, after which the animal was returned to its home cage for 1 h. After the inter‐trial interval, session 2 involved the same mouse freely exploring the testing arena. One of the previous objects was replaced with a novel object of a different shape and color for 10 min. A “discrimination index” was calculated by subtracting the time the animal spent exploring the familiar object from the time spent exploring the novel object, which was then divided by the total time spent exploring both of the objects.

#### 2.6.4 Social Interaction

Social interaction (SI) test was conducted on PND 25 to assess changes in social behavior as previously described (Ratnayake et al. 2014; Ratnayake et al. 2014). The apparatus consisted of an open‐top rectangular box of 60 × 30 × 45 cm (L × W × H) divided into three chambers of 20 × 30 cm (L × W), with an 8 × 8 cm square opening cut into the internal walls to allow the mouse to move between chambers. For reference, the chambers were numbered 1–3 from left to right. Chambers 1 and 3 contained large cylinders measuring 40 × 8 cm (H × D), where a “stranger,” age‐ and sex‐matched spiny mouse could be placed. The cylinder was drilled with numerous 0.6‐cm diameter holes to allow for visual, tactile, and olfactory contact through the enclosure. The social interaction tests consisted of a spiny mouse being placed in chamber 2 and allowed habituation to the social interaction apparatus for 5 min, after which the spiny mouse was taken out and the stranger spiny mouse placed in the cylinder enclosure in chamber 1 or 3. The test spiny mouse was placed back in the middle of chamber 2, and the duration of interaction of the test spiny mouse with the stranger was recorded. A “discrimination index” was calculated by subtracting the time the animal spent exploring the stranger mouse from the time spent not exploring, which was then divided by the total time spent exploring. A positive discrimination index indicated a preference for exploring the stranger mouse, 0 indicated no preference for either, and a negative discrimination index indicated a preference for not exploring the stranger mouse.

#### 2.6.5 Elevated plus Maze

Elevated plus maze (EPM) test was conducted on PND 45 to assess anxiety and impulsiveness behavior (Walf and Frye 2007) as previously described (Ratnayake et al. 2014; Ratnayake et al. 2014; Tran et al. 2025). The apparatus consisted of a plus‐shaped maze raised 50 cm above the ground, with four arms extending from a central 10 × 10 cm platform with two opposing open arms (40 × 10 cm) and two opposing closed arms (also 40 × 10 cm) surrounded by 15‐cm high walls. At the start of the test, the mouse was placed in the center facing an open arm, then allowed to freely explore the maze for 5 min. Activity was recorded using an overhead camera located above the apparatus. A “discrimination index” was calculated by subtracting the time the animal spent exploring the closed arms from the time spent not exploring, which was then divided by the total time spent exploring.

#### 2.6.6 Prepulse Inhibition

Prepulse inhibition (PPI) test was conducted on PND 27 to assess sensorimotor gating using the SR‐LAB startle apparatus (San Diego Instruments, San Diego, Calif., USA) as previously described (Ratnayake et al. 2014; Ratnayake et al. 2014). Each test began with 2 min of apparatus acclimatization, followed by five consecutive pulse‐alone (PA) trials to habituate the animal and establish a stable startle response. The animals were then presented with a total of 35 pseudorandom trials consisting of 10 interspersed PA trials, five no‐stimulus trials (i.e. background noise; NS), and 20 prepulse‐pulse trials, where a prepulse sound of 2, 4, 6, 8, 12, or 16 dB above background (70 dB) and 20 ms duration were presented 100 ms before the startle pulse. The acoustic startle pulse was a white‐noise signal at 115 dB, with a duration of 40 ms. The session was concluded with five consecutive PA trials. The interval between successive trials was ∼20 s.

The average PPI was expressed as relative inhibition of the startle response due to the prepulse, that is, %PPI = (amplitude of startle pulse − (amplitude of each prepulse + startle pulse))/(amplitude of startle pulse) × 100%. For each animal, mean startle amplitudes were calculated separately for the interspersed PA trials and for prepulse‐plus‐pulse trials at each prepulse intensity before calculating %PPI. A %PPI was calculated for all of the prepulse intensities. A higher %PPI score indicates a greater reduction in startle magnitude in prepulse + pulse trials relative to that in PA trials. The average startle at the start and at the end of the PPI test was also recorded and expressed as the average amplitude of the five startle PA trials (mV) to assess habituation of the acoustic startle response.

### 2.7 Offspring Post‐Mortem Weights and Organ Collection

On PND 45, the spiny mice were anaesthetized via isoflurane overdose ∼95% in room air. Body weights were recorded before euthanasia via decapitation. The offspring liver, thymus, brain, and spleen were dissected and weighed. All organ weights are presented as % body weight (BW).

### 2.8 Statistical Analyses

Statistical analyses for the acute response study, including maternal organ weights, gene transcription, and cytokine data, were performed using a one‐way analysis of variance (ANOVA) with significant outcomes followed by Tukey's multiple comparisons test for parametric data or a Kruskal‐Wallis test with significant outcomes followed by Dunn's multiple comparisons test for non‐parametric data. These analyses were performed using GraphPad Prism (version 10.2; GraphPad Software Inc., CA, USA).

For the long‐term assessment of offspring outcomes, statistical analyses were conducted using SPSS (version 27; IBM Corporation, 2020). Postnatal growth and behavioral tests completed across multiple trials (rotarod), postnatal ages (OFT), or repeated stimulus conditions (PPI) were analyzed using repeated measures analysis of covariance (ANCOVA), with sex and litter size included as covariates. Within‐subject factors were trial, postnatal age, prepulse intensity, or time (start, interleaved or end PA responses), as appropriate. Behavioral tests conducted at a single postnatal age and post‐mortem assessments were analyzed using one‐way ANCOVA, with sex and litter size included as covariates. When overall significance was obtained, a Bonferroni post hoc test was conducted. All graphs were made using GraphPad Prism (version 10.2; GraphPad Software Inc.).

## 3 Results

### 3.1 Acute Response to Poly(I:C) and Rytvela Administration

During the 8 h after the initiation of the Poly(I:C) and Poly(I:C) + rytvela dosing regimens at mid‐gestation, no differences in maternal body weight or change in body temperature were observed in spiny mouse dams (Table 1). After the second dose, 1/10 Poly(I:C) dams displayed sluggish behavior and 5/10 Poly(I:C) dams displayed vaginal bleeding. Control and Poly(I:C) + rytvela dams did not exhibit abnormal behavior or vaginal bleeding. No change in maternal care was observed for any group.

**TABLE 1:** Acute maternal physiological responses and outcomes to Poly(I:C) and rytvela administration at mid‐gestation.

Maternal responses and outcomes	Control (*n* = 10)	Poly(I:C) (*n* = 10)	Poly(I:C) + rytvela (*n* = 10)	ANOVA or Kruskal–Wallis *p* value
Maternal body weight (g)	50.14 ± 3.00	48.81 ± 3.39	48.82 ± 2.64	*F*(2, 27) = 0.38, *p* = 0.34
Basal body temperature (°C)	36.89 ± 0.42	36.36 ± 0.54	36.59 ± 0.55	*F*(2, 25) = 0.08, *p* = 0.08
Δ Body temperature (2 h after first injection)	−0.42 ± 0.52	−0.46 ± 0.77	−0.14 ± 0.41	*H*(2) = 1.68, *p* = 0.43
Any febrile response	0/10	0/10	0/10	—
Abnormal behavior (reduced alertness)	0/10	1/10	0/10	—
Vaginal bleeding	0/10	5/10	0/10	—
Litter size	3.5 ± 0.71	3.6 ± 0.97	2.8 ± 0.42^#^	*H*(2) = 7.32, *p* = 0.03*
Brain weight (% BW)	1.67 ± 0.11	1.73 ± 0.15	1.68 ± 0.10	*F*(2, 27) = 0.23, *p* = 0.77
Liver weight (% BW)	3.46 ± 0.25	3.03 ± 0.16^*^	3.05 ± 0.15^*^	*H*(2) = 14.66, *p* = 0.0007^***^
Spleen weight (% BW)	0.20 ± 0.07	0.23 ± 0.05	0.22 ± 0.02	*H*(2) = 3.81, *p* = 0.15
Thymus weight (% BW)	0.03 ± 0.01	0.04 ± 0.01^*^	0.03 ± 0.01^*^	*F*(2, 27) = 0.43, *p* = 0.009^**^
Fetal outcomes	Control (*n* = 35)	Poly(I:C) (*n* = 36)	Poly(I:C) + Rytvela (*n* = 28)	Kruskal–Wallis *p* value
Placenta:fetal weight ratio	0.69 ± 0.10	0.70 ± 0.19	0.74 ± 0.15	*H*(2) = 2.11, *p* = 0.35

*Note*: Data are shown as mean ± SD and incidence. Data were analyzed by one‐way ANOVA for parametric data and a Kruskal–Wallis test for non‐parametric data; significance was followed up by Tukey's or Dunn's multiple comparisons test, respectively. For pair‐wise comparisons: ^*^significantly different to control and ^#^significantly different to Poly(I:C).

*p<0.05, **p<0.01, ***p<0.001

Abbreviation: BW, body weight.

Pregnancy maintenance and fetal and maternal outcomes were analyzed at post‐mortem, 8 h after the first administration of treatments (Table 1). There was no sign of fetal resorption or overt abnormalities in any group. There were no differences in maternal brain or spleen weight between treatment groups. Liver weight was less (∼12%) in both the Poly(I:C) and Poly(I:C) + rytvela compared to controls (H(2) = 7.317, P = 0.03; p = 0.003 and 0.002, respectively). In contrast, the thymus weight of Poly(I:C) dams was greater than that of controls and Poly(I:C) + rytvela (F(2,27) = 5.715, P = 0.01; p = 0.02 and 0.02 respectively). The placenta:fetal weight ratio was not different between the groups.

### 3.2 IL‐1β Protein Analysis

**FIGURE 2:**
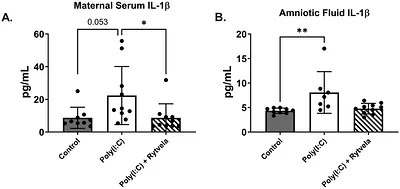
IL‐1β concentrations in (A) maternal serum and (B) amniotic fluid, 8 h after first administration of either Poly(I:C), Poly(I:C) + rytvela, or saline‐vehicle (n = 10 dams/treatment group). Data are shown as mean ± SD. Data were analyzed by Kruskal–Wallis and Dunn's multiple comparisons post hoc test. *p < 0.05; **p < 0.01.

IL‐1β protein was higher in amniotic fluid (H(2) = 9.768, P = 0.008; p = 0.006) but was not statistically higher in maternal serum (H(2) = 8.992, p = 0.01; p = 0.053) in Poly(I:C) dams compared to controls (Figure 2A,B). Poly(I:C) + rytvela dams had significantly lower IL‐1β protein in maternal serum compared to the Poly(I:C) alone group (Figure 2A; p = 0.02). In the amniotic fluid of Poly(I:C) + rytvela dams, IL‐1β was not different from controls or Poly(I:C) alone (Figure 2B; p > 0.1).

### 3.3 Gene Expression Analysis

Pro‐inflammatory gene expression was measured in gestational tissues and maternal spleen and thymus recovered at autopsy (Table 2). In the uterus, the expression of Il1b, Il6, and Tnf remained constant across the groups. In the placental labyrinth, Il1b, Il6, and Tnf expression were increased in both the Poly(I:C) and Poly(I:C) + rytvela groups compared to controls (p < 0.01). In the placental junctional zone, Tnf expression was reduced in the Poly(I:C) alone compared to the control (p = 0.03) but not Poly(I:C) + rytvela group. Il1b and Il6 expression was not different between the groups.

**TABLE 2:** Gene expression in gestational and maternal immune tissues.

Tissue	Gene of Interest	Control (*n* = 10)	Poly(I:C) (*n* = 10)	Poly(I:C) + rytvela (*n* = 10)	*Kruskal‐Wallis P value*
Pregnant uterus	*Il1b*	0.39 (0.05, 1.71)	2.12 (0.87, 7.39)	1.43 (0.10, 3.66)	*H*(2) = 4.13, *p* = 0.13
	*Il6*	0.80 (0.15, 1.78)	1.66 (0.54, 2.83)	0.37 (0.10, 4.31)	*H*(2) = 1.94, *p* = 0.38
	*Tnf*	0.76 (0.20, 2.06)	1.13 (0.45, 4.37)	0.39 (0.12, 2.24)	*H*(2) = 1.93, *p* = 0.38
Placenta (labyrinth)	*Il1b*	0.16 (0.04, 1.45)	3.72 (1.93, 16.5)^*^	11.0 (2.80, 19.3)^*^	*H*(2) = 10.93, *p* = 0.004^**^
	*Il6*	0.23 (0.02, 2.14)	4.02 (2.25, 6.03)^*^	5.43 (2.45, 19.72)^*^	*H*(2) = 13.03, *p* = 0.002^**^
	*Tnf*	0.75 (0.48, 1.58)	2.47 (1.85, 3.39)^*^	3.45 (1.48, 5.00)^*^	*H*(2) = 11.98, *p* = 0.003^**^
Placenta (junctional zone)	*Il1b*	0.35 (0.12, 1.20)	0.15 (0.09, 0.20)	0.80 (0.15, 4.46)	*H*(2) = 5.53, *p* = 0.06
	*Il6*	0.20 (0.05, 1.57)	0.06 (0.03, 0.12)	0.26 (0.03, 0.38)	*H*(2) = 3.81, *p* = 0.15
	*Tnf*	0.97 (0.28, 1.43)	0.20 (0.08, 0.25)^*^	0.47 (0.21, 0.86)	*H*(2) = 6.92, *p* = 0.03^*^
Maternal spleen	*Il1b*	0.57 (0.12, 1.31)	4.66 (2.83, 25.3)^*^	23.4 (9.90, 69.6)^*^	*H*(2) = 18.79, *p* < 0.001^**^
	*Il6*	0.44 (0.13, 2.09)	8.50 (2.91, 37.5)^*^	59.5 (28.4, 102.0)^*^	*H*(2) = 18.66, *p* < 0.001^**^
	*Tnf*	0.74 (0.40, 1.23)	2.93 (1.75, 3.89)^*^	7.02 (2.98, 13.8)^*^	*H*(2) = 22.45, *p* < 0.001^**^
Maternal thymus	*Il1b*	0.34 (0.14, 1.51)	0.47 (0.18, 1.40)	3.84 (2.27, 6.34)^*#^	*H*(2) = 11.04, *p* = 0.004^**^
	*Il6*	0.33 (0.06, 2.00)	0.37 (0.21, 0.60)	9.49 (4.00, 18.3)^*#^	*H*(2) = 12.87, *p* = 0.002^**^
	*Tnf*	0.79 (0.23, 1.38)	0.40 (0.23, 1.13)	3.83 (1.64, 6.61)^*#^	*H*(2) = 9.38, *p* = 0.009^**^

*Note*: Data are expressed as median and IQR. Data were analyzed by Kruskal‐Wallis with Dunn's multiple comparisons test. Superscript symbols indicate significant differences between treatment groups based on post hoc analysis; for pair‐wise comparisons: ^*^significantly different to Control and ^#^significantly different to Poly(I:C). p < 0.05 was deemed significant.

**p<0.01

The most overt changes in gene expression were seen in the maternal spleen and thymus. In the spleen, Il1b (p < 0.013) and Il6 (p < 0.001) levels were significantly higher after Poly(I:C) and Poly(I:C) + rytvela treatments compared to controls. Tnf (p < 0.001) also increased in both the Poly(I:C) and Poly(I:C) + rytvela groups versus controls, but to a lesser degree. In the thymus, Poly(I:C) alone did not change gene expression from controls; however, in the Poly(I:C) + rytvela group, Il1b (p < 0.004), Il6 (p < 0.02), and Tnf (p < 0.02) levels increased compared to both the control and Poly(I:C) alone groups.

### 3.4 Offspring Growth

Four litters per treatment group were allowed to progress to term, and offspring outcomes were analyzed in seven pups in the saline‐control group, eight pups in the Poly(I:C) group, and 11 pups in the Poly(I:C) + rytvela group. The percentage of male pups born in the Poly(I:C) group (37.5%; *n*(male:female) = 3:5) was slightly lower than saline controls (43%; 3:4) and the Poly(I:C) + rytvela (45%; 5:6) cohort. As such, both litter size and sex were included as covariates in all statistical analyses.

**FIGURE 3:**
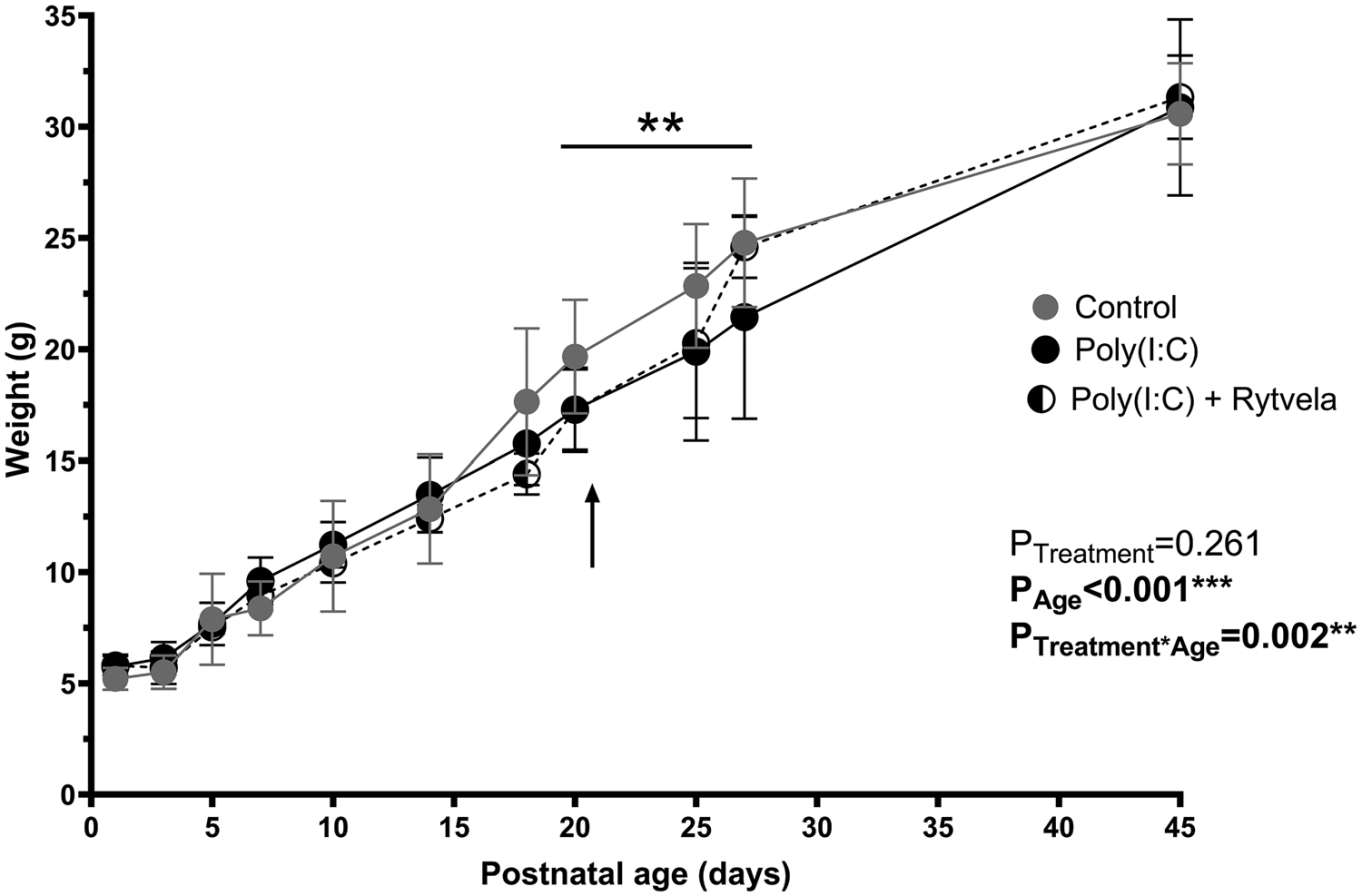
Weight gain trajectory of spiny mice offspring over the 45‐day behavioral testing schedule. Data are shown as mean ± SD per treatment group; control: *n* = 7; Poly(I:C): *n* = 8; Poly(I:C) + rytvela: *n* = 11. Statistical analysis repeated measures ANCOVA to account for covariates (sex and litter size). Main effects were assessed by post hoc Bonferroni tests. *p* < 0.05 was deemed significant. **denotes time period where significant differences between treatment groups were identified. Main effects: **p < 0.01; ***p < 0.001. The arrow denotes when pups were weaned (PND 21).

Across the entire 45‐day testing period, weight progressively increased with age for all pups (Figure 3; *F*(2.53, 53.12) = 7.95, *P*_age_ < 0.001). At birth (PND 1) to PND 18, there were no significant differences in the pup weights between the experimental groups. There were, however, significant differences in body weight at PND 20 and PND 25, where growth rate slowed in Poly(I:C) and Poly(I:C) + rytvela exposed pups. The Poly(I:C) + rytvela exposed pups returned to the same growth trajectory as controls from PND 27; however, growth rate did not recover in the Poly(I:C) exposed pups until PND 45. Overall, weight gain was consistent for both male and female pups (*F*(1, 21) = 3.56, *P*_sex_ < 0.073). There were no significant differences in body or organ weights (brain, liver, thymus, and spleen) recorded at PND 45 (Table S4).

### 3.5 Offspring Behavior

#### 3.5.1 OFT

**FIGURE 4:**
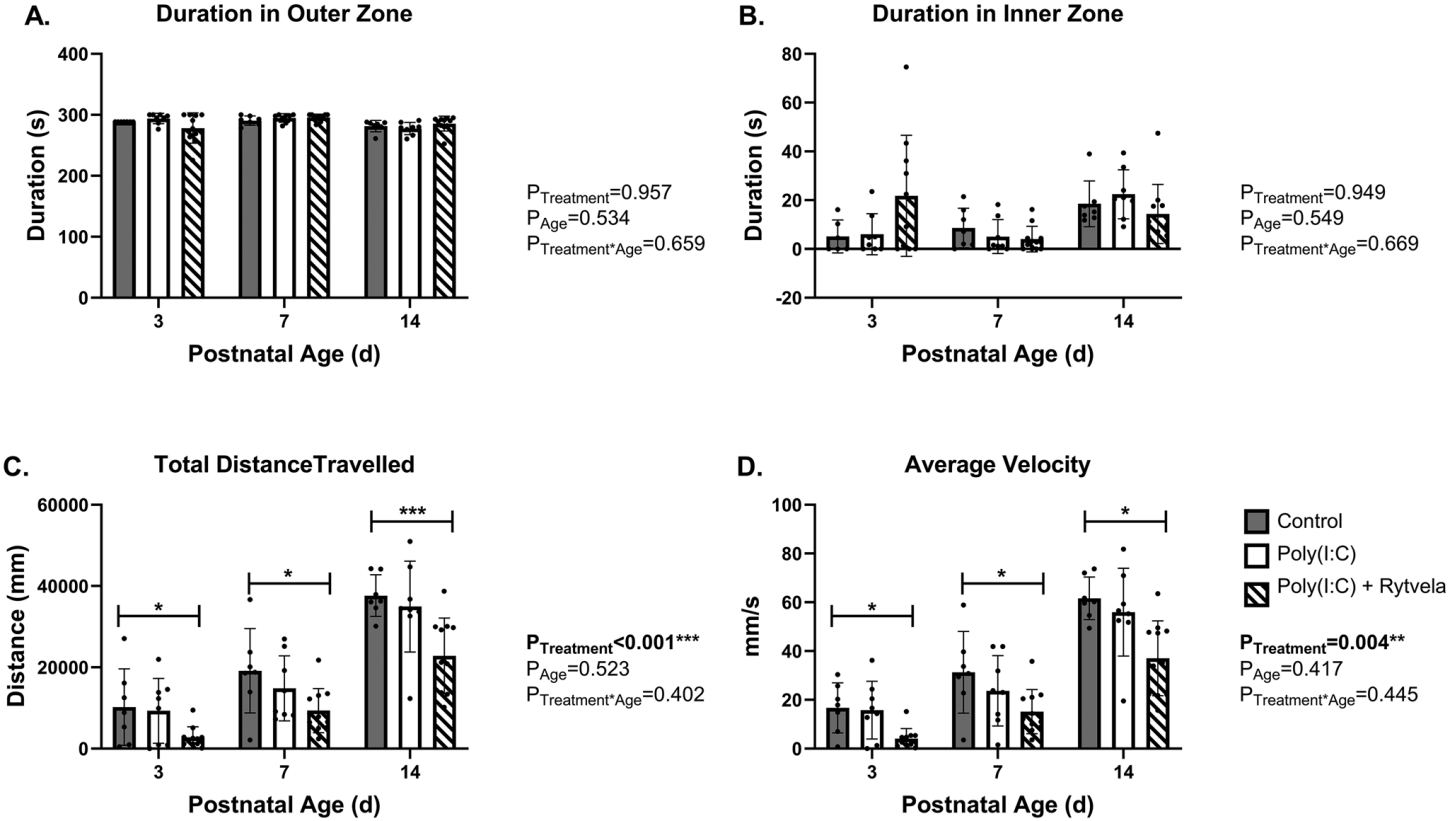
(A) Duration of time spent in the outer zone of the arena, (B) duration of time spent in the inner zone of the arena, (C) total distance travelled, and (D) average velocity from open field testing at PND 3, 7, and 14. Data are presented as mean ± SD. Data were analyzed by repeated measures ANCOVA to account for covariates (sex and litter size) and post hoc Bonferroni test. * denotes significant differences between treatment groups on post hoc analysis. Control: *n* = 7; Poly(I:C): *n* = 8; Poly(I:C) + rytvela: *n* = 11. Main effects: ** *p* <0.01; ***p < 0.001.

Irrespective of prenatal treatment, in the open field test, the spiny mice offspring showed a preference for the outer zone of the arena, mostly circumventing the inner zone (Figure 4A,B). Pups born to dams treated with Poly(I:C) did not exhibit any behavior change compared to controls. In contrast, those of Poly(I:C) + rytvela‐treated dams showed a ∼52% reduction in total distance travelled (Figure 4C, *F*(2,21) = 15.58, *P*_Treatment_ < 0.001; *p* < 0.001), and reduced average velocity of movement compared to controls, across all postnatal ages examined (Figure 4D, *F*(2,21) = 7.42, *P*_Treatment_ = 0.004; *p* = 0.021).

#### 3.5.2 Rotarod

**FIGURE 5:**
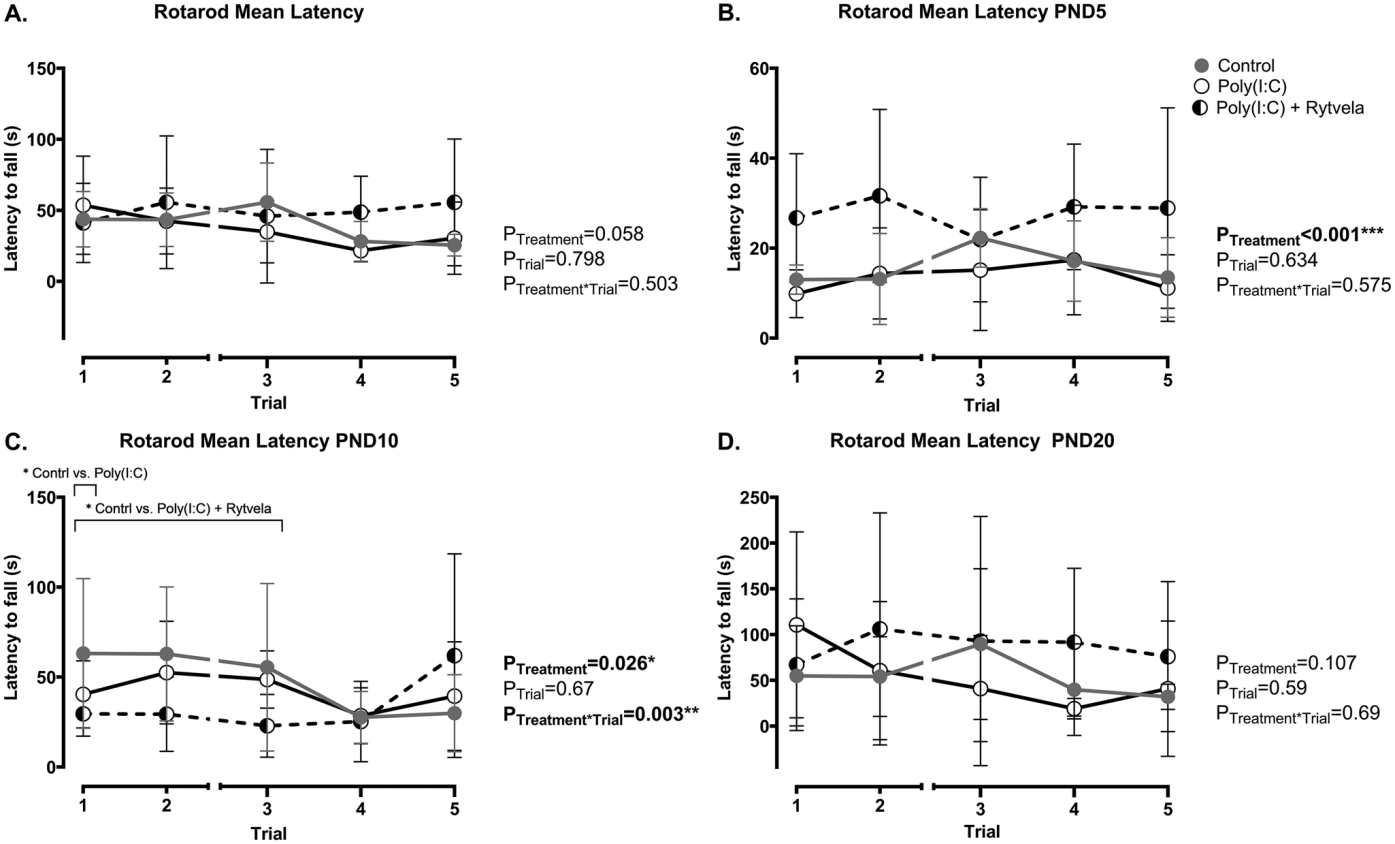
Latency to fall off the rotarod is presented as mean latency at (A) all ages (averaged across all ages), (B) PND 5, (C) PND 10, and (D) PND 20. Data are presented as mean ± SD. Data were analyzed by repeated measures ANCOVA to account for covariates (sex and litter size). The break in the *x*‐axis signifies a 10‐min rest period between trials 2 and 3. Control: *n* = 7; Poly(I:C): *n* = 8; Poly(I:C) + rytvela: *n* = 11. * denotes significant differences between treatment groups on post hoc analysis. Main effects: *p < 0.05; **p < 0.01; ***p < 0.001.

There was a trend toward a treatment effect on mean latency at all ages combined (Figure 5A; *F*(2,21) = 3.27, *P*_Treatment_ = 0.058). At PND5, the Poly(I:C) + rytvela group remained on the rotarod for longer compared to the Poly(I:C) group (Figure 5B; *F*(2,20) = 10.71, *P*_Treatment_ < 0.001; *p* < 0.001) but was not statistically different from controls (*p* = 0.092). At PND 10, there was a significant interaction effect of treatment and trial on rotarod latency (Figure 5C; *F*(8,84) = 3.18, *P*_Treatment_ = 0.003) in which control pups had higher rotarod latency compared to the Poly(I:C) and Poly(I:C) + rytvela group during trials 1–3 (*p* < 0.02). However, by PND 20, no differences in latency to fall were found across treatment groups and trials (Figure 5D; *P*_Age_ and *P*_Treatment_ > 0.05).

#### 3.5.3 Novel Object Recognition Test, Social Interaction, and Elevated Plus Maze

**FIGURE 6:**
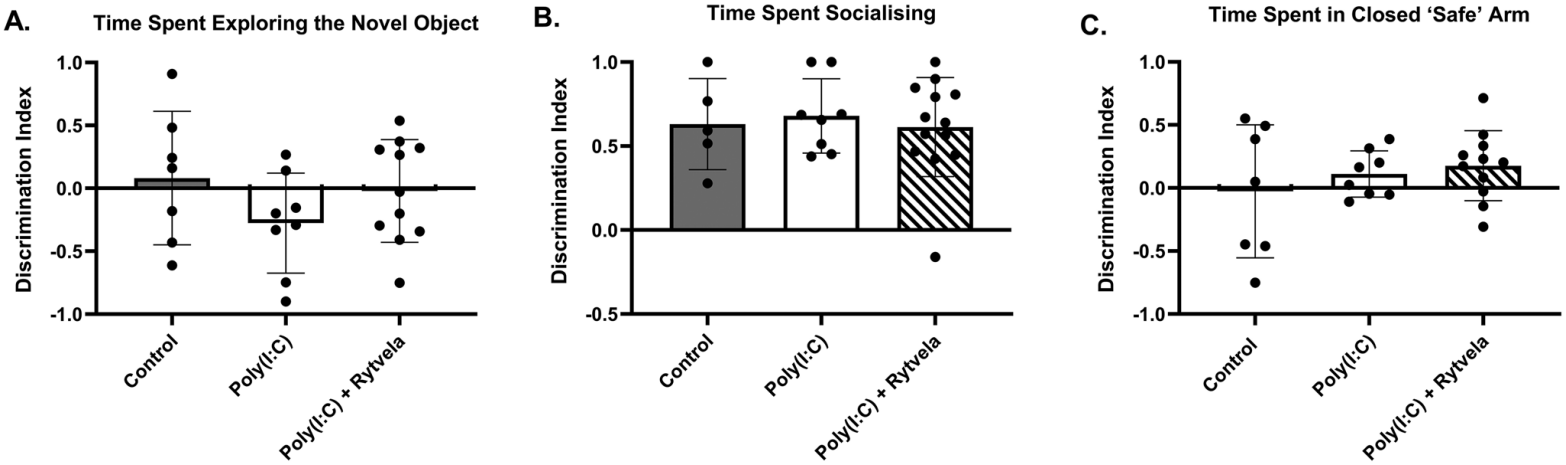
Offspring behavioral testing discrimination index of (A) novel object recognition at PND 18 for the novel object, (B) social interaction at PND 25 for the stranger mouse and, (C) elevated plus maze at PND 45 for time spent in the closed “safe” arm. Data are presented as mean ± SD. Data were analyzed by ANCOVA to account for covariates (sex and litter size). Control: *n* = 7; Poly(I:C): *n* = 8; Poly(I:C) + rytvela: *n* = 11.

There was no significant effect of treatment group on time spent actively examining a novel object (Figure 6A; *F*(2,20) = 1.07, *P*_Treatment_ = 0.36). When social interactions were examined at PND 21, there was no difference in the time spent with the “stranger” mouse relative to the empty chambers of the arena between groups (Figure 6B; *F*(2,20) = 0.13, *P*_Treatment_ = 0.129). Finally, all pups, irrespective of treatment group, preferred to spend time in the closed arms of the EPM at PND 45 (Figure 6C; *F*(2,20) = 1.67, *P*_Treatment_ = 0.21).

#### 3.5.4 Prepulse Inhibition

**FIGURE 7:**
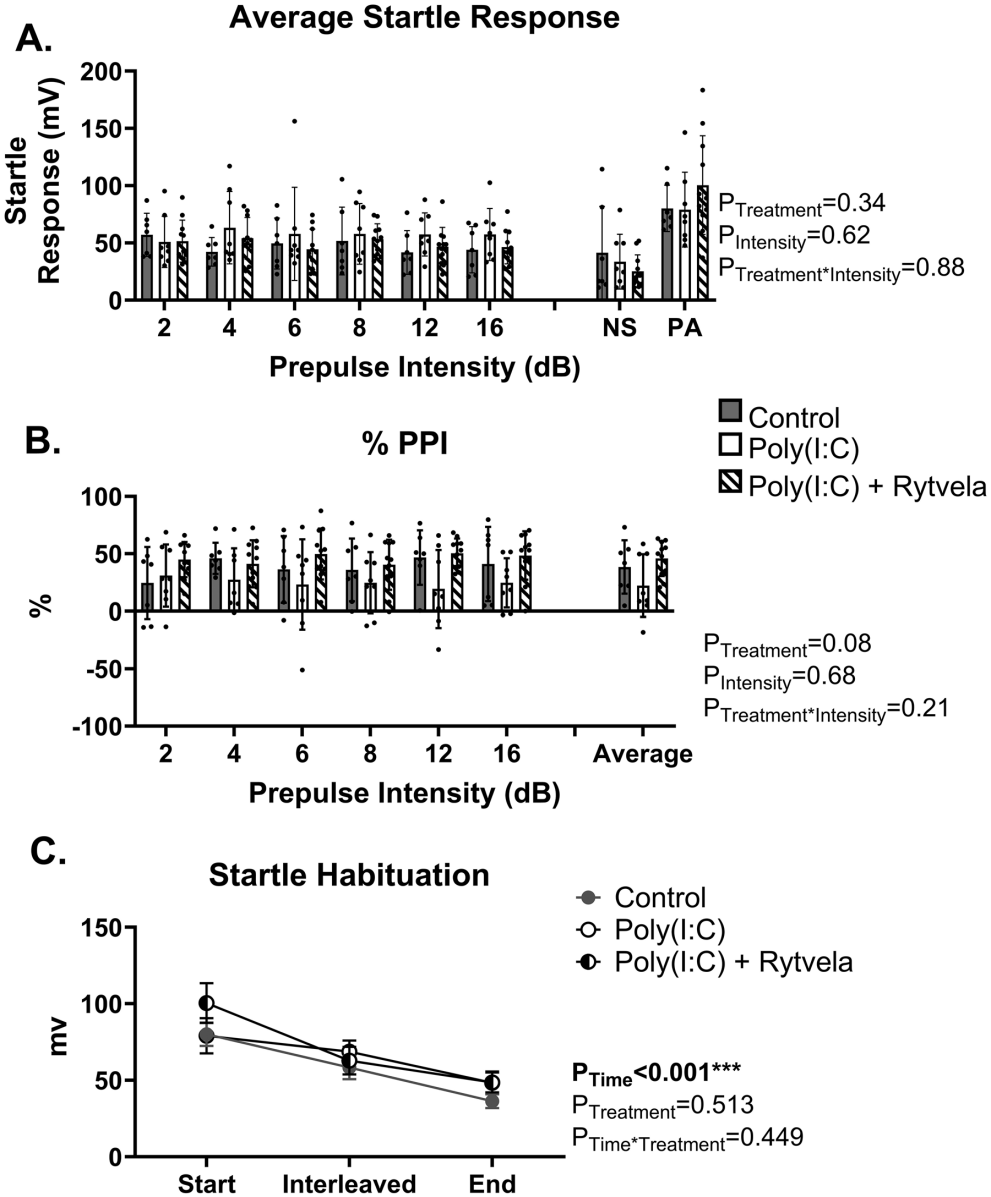
Outcome of prepulse inhibition testing at PND 27. (A) The average startle response to prepulse intensities (dB), no stimulation (NS) and pulse‐alone (PA) trials, (B) % prepulse inhibition (PPI) at each prepulse intensity and average percentage, and (C) startle habituation across time (start, interleaved, and end) of PPI trial. Data are presented as mean ± SD. Data were analyzed by ANCOVA to account for covariates (sex and litter size) and post hoc Bonferroni test. Control: *n* = 7; Poly(I:C): *n* = 8; Poly(I:C) + rytvela: *n* = 11. Main effects: ***p < 0.001.

There was no significant impact of treatment group on the average startle responses across prepulse intensities ranging from 2 to 16 dB (Figure 7A; *F*(2,23) = 1.13, *P*_Treatment_ = 0.34). Overall, there were no differences between the treatment groups in the %PPI across the six prepulse intensities or in the average %PPI response (Figure 7B; *F*(2,23) = 2.77, *P*_Treatment_ = 0.084). PA startle responses decreased across the testing session in all treatment groups, demonstrating normal habituation to repeated acoustic stimuli (Figure 7C). A significant main effect of session stage (start, interleaved, and end PA trials) was observed (*F*(2,46) = 19.54, *P*_Time_ < 0.0001), with no main effect of treatment group (*F*(2,23) = 0.69, *P*_Treatment_ = 0.513).

## 4 Discussion

This study used the precocial spiny mouse to assess the effect of co‐administering the IL‐1R antagonist rytvela together with a mid‐gestation maternal challenge induced by the TLR3 agonist and viral mimetic Poly(I:C). The primary outcomes evaluated were acute maternal and placental inflammatory responses and developmental and behavioral outcomes in offspring. Exposure to Poly(I:C) increased IL‐1β cytokine protein levels in maternal serum and amniotic fluid 8 h after the initial insult, which was diminished with the co‐administration of rytvela. Poly(I:C) also elevated the expression of *Il1b, Il6*, and *Tnf* genes in the placenta and maternal spleen, indicating a systemic response, which rytvela did not mitigate. In the maternal thymus, the combined treatment of Poly(I:C) and rytvela increased *Il1b, Il6*, and *Tnf* expression to a greater extent than that observed in the Poly(I:C) alone treatment group. Growth and behavioral analyses revealed that offspring exposed to Poly(I:C) at mid‐gestation had an altered growth trajectory, which was partially corrected by rytvela co‐administration, resulting in an earlier return to control growth trajectories during postnatal development. Our Poly(I:C) treatment did not produce measurable behavioral deficits under the conditions of this study. However, PND 3–14 pups exposed to Poly(I:C) + rytvela had lower total locomotor activity observed in the open field test. Overall, in utero exposure to rytvela effectively modulated maternal IL‐1β and partially mitigated the negative effects of Poly(I:C) on offspring growth; however, the treatment was associated with potentially adverse maternal inflammatory parameters and early postnatal locomotor activity.

### 4.1 Altered Maternal Immune Response to Poly(I:C) With Rytvela Treatment

In this study, the maternal immune response 8 h after Poly(I:C) administration at mid‐gestation included an upregulation of pro‐inflammatory mediators, but no signs of a maternal febrile response or malaise. This is consistent with previous studies in spiny mice (Ratnayake et al. 2012). While preterm birth (PTB) is not a characterized feature of a Poly(I:C)‐induced maternal immune challenge at this mid‐gestation time point in spiny mouse or conventional laboratory mouse strains (Arsenault et al. 2014), vaginal bleeding was noted in 50% of Poly(I:C) treated dams. This was not observed in dams when Poly(I:C) was co‐administered with rytvela. In pregnant CD‐1 mice challenged with either lipopolysaccharide (LPS; 10 mg IP) or IL‐1β (1 mg/kg intrauterine) in studies designed to elicit PTB, rytvela reduced the incidence of PTB by 60%–70% in a dose‐dependent manner (Habelrih et al. 2023). Rytvela's capacity to inhibit proinflammatory cytokine upregulation in gestational tissues acts to suppress the production of uterine activation proteins, thereby preventing preterm labor (Quiniou et al. 2008; Nadeau‐Vallée et al. 2015). Indeed, in the current study, co‐administration of rytvela with Poly(I:C) significantly reduced IL‐1β protein levels in maternal serum and amniotic fluid 8 h after treatment, suggesting a dampening of the inflammatory response to the Poly(I:C) challenge. However, this was not consistently apparent at a transcriptional level. While elevated pro‐inflammatory gene expression was abrogated in some tissues with co‐administration of rytvela, it was amplified in others.

In light of these ambiguous findings, the limitations of the transcriptional data need to be acknowledged. First, the 8‐h time point of analysis was considerably later than the usual peak of cytokine gene expression, which generally occurs around 2–6 h after the induction of an inflammatory response (Arrode‐Brusés and Brusés 2012). Second, our expression data showed substantial between‐animal variation, implying that larger sample sizes would be required for confident interpretation. Finally, the possibility of species differences in IL‐1R signaling and gene expression cannot be discounted. Transcriptional programs regulated by IL1R signaling may have differences in spiny mice compared to those in *Mus musculus*, where most previous rytvela studies have been undertaken.

Nevertheless, our data suggest that while rytvela can reduce levels of IL‐1β, it may exacerbate other pro‐inflammatory cytokines in response to the viral mimetic Poly(I:C). Interestingly, increased expression of *Il1b*, *Il6*, and *Tnf* occurred only in the thymus of rytvela‐ and Poly(I:C)‐exposed dams, with a compounded increased expression within their spleens suggesting an exacerbated pro‐inflammatory response.

This is in contrast to rytvela's capacity to suppress cytokine expression following immune challenges elicited by IL‐1, LPS, or heat‐killed *E. coli* in laboratory mice—the latter two of which are mediated via TLR4 signaling (Jiang et al. 2003) through an adaptor protein known as TIR‐domain‐containing adapter inducing interferon (TRIF), which elicits the expression of type 1 interferons (IFNs) and inflammatory cytokines, including components that do not depend on interleukin‐1 signaling for their pro‐inflammatory effects (Jiang et al. 2003; Ullah et al. 2016). Rytvela is a non‐competitive IL‐1R antagonist, and this may account for its reduced efficacy in suppressing pro‐inflammatory cytokine transcription after Poly(I:C) treatment compared to other studies of rytvela that utilize TLR2 or TLR4 stimulants (Jiang et al. 2003), which signal through different adaptor proteins and signaling pathways (Dowling and Mansell 2016). However, even if rytvela was less effective in modulating the response elicited via TLR3, the amelioration of vaginal bleeding and some inflammatory mediators implies at least some degree of modulation of the inflammatory cascade afforded by rytvela when co‐administered with Poly(I:C).

### 4.2 The Effects of Poly(I:C) and Rytvela Exposure on Offspring Growth and Behavior

While mid‐gestation Poly(I:C) administration had no overt effect on birth weight or perinatal survival, an altered postnatal growth trajectory was noted between PND 18–27, where the growth of both Poly(I:C) and Poly(I:C) + rytvela offspring slowed in comparison to their control counterparts. Rytvela‐exposed offspring recovered from this lag faster than the pups exposed to Poly(I:C) without rytvela. The mechanisms and significance of this slowed growth remain unknown. However, it is worth noting that the timing of recovery coincides with the age of self‐weaning for spiny mice (Haughton et al. 2016), raising the possibility that effects of treatments on maternal lactation contribute to the underlying mechanism.

The Poly(I:C) dosing regimen used in the current study, optimized to produce a robust maternal IL‐1β response without causing excessive fetal loss, did not result in significant changes in offspring behavior up to PND 45. This is in contrast to previous spiny mouse and conventional rodent studies, which have reported negative changes in social interaction, memory and learning, and PPI (Ratnayake et al. 2014; Ratnayake et al. 2012). The absence of significant behavioral abnormalities in the present study is consistent with increasing evidence that offspring behavioral responses to maternal Poly(I:C) are variable and depends on the dose and formulation of Poly(I:C), route and gestational timing of administration, the magnitude of the maternal immune response, genetic and breeder background, sex, behavioral domain assessed, and laboratory environment (Bucknor et al. 2022; Otero and Antonson 2022; Haddad et al. 2020). Consequently, the ability of rytvela to ameliorate behavioral deficits following maternal immune activation could not be adequately evaluated in the present study. Future studies in which neurobehavioral outcomes are established as the primary endpoint and are powered accordingly will be required to determine whether prenatal IL‐1β modulation with rytvela provides meaningful behavioral benefit.

### 4.3 Translational Potential of Rytvela to Reduce Maternal Immune Activation

While IL‐1β has been implicated in driving in utero inflammation and behavioral deficits, complete suppression of all IL‐1R‐induced signaling pathways can have a detrimental impact on offspring behavior and development, given the important neuroendocrine function of IL‐1 and its role in regulating neurogenesis, synaptic plasticity, learning, memory, and cognition (Jones et al. 2015). If proven to be safe and efficacious, the allosteric nature of rytvela and its selective capacity to inhibit p38 MAPK and ROCK2 signaling while preserving the NF‐κB signaling pathway (Nadeau‐Vallée et al. 2015) may make it a highly useful anti‐inflammatory therapeutic with important neurodevelopment‐sparing properties.

This study provides the first data on the postnatal effects of in utero rytvela exposure. Overall, rytvela administered at mid‐gestation protected against some Poly(I:C)‐induced effects and did not cause any significant adverse impacts on offspring health or welfare. However, we consistently observed a decline in gross locomotor activity and velocity at multiple postnatal ages, associated with the co‐administration of Poly(I:C) and rytvela, through open‐field testing. A qualitative review of the open field trials links these decreases to a higher degree of sedentary and freezing behaviors, indicative of reduced motivation or fear conditioning (Gouveia and Hurst 2017; Valentinuzzi et al. 1998), as opposed to supported or unsupported rearing, which are more indicative of stress and anxiety (Sturman et al. 2018). The overall significance of this finding remains unclear, given it was not linked to altered body composition, motor coordination, or social behaviors. Future studies should aim to elucidate the underlying mechanisms and long‐term behavioral consequences of the reduced locomotor activity.

### 4.4 Limitations and Future Directions

A key limitation of the study was the relatively small number of litters entered into the behavioral testing regimen. This likely reduced our ability to detect significant effects for some of the tests, most notably the mean latency outcome of rotarod testing. In addition, while offspring sex was included as a covariate in statistical comparisons of offspring outcomes, the study was not powered to assess male and female offspring independently. This is noteworthy, as previous studies assessing rodent behavioral outcomes following in utero Poly(I:C) exposure have noted more pronounced deficits in female offspring (Arsenault et al. 2014; Su et al. 2022). We also recognize that the absence of data on the effects of rytvela administration without Poly(I:C) confounds our ability to determine whether effects seen in the Poly(I:C) + rytvela group are due to rytvela acting independently or interactions between Poly(I:C) and rytvela. While it is unlikely that rytvela would be clinically administered without confirmed infection or inflammation and elevated IL‐1β, future studies assessing the effects of rytvela alone will further the understanding of its safety profile.

This is the first preclinical study to assess offspring behavior following rytvela administration in utero. While this study provides evidence that prenatal rytvela exposure can attenuate aspects of the maternal inflammatory response and improve postnatal growth following Poly(I:C) challenge, its effects on offspring neurobehavioral outcomes remain uncertain. The spiny mouse model could be utilized to provide further efficacy data on the capacity of rytvela to mitigate adverse outcomes of maternal inflammation during pregnancy, particularly those associated with fetal inflammatory injury and preterm birth. For example, challenging the spiny mouse model with pro‐inflammatory agents that activate the TLR2 or TLR4 pathways may provide further insight into rytvela's efficacy in dampening adverse developmental outcomes arising after bacterial infection during pregnancy. Contemporaneous studies conducted in CD‐1 mice suggest that a minimum treatment duration of 36 h with rytvela is required to improve fetal outcomes (Habelrih et al. 2023). Despite the slightly higher dose of rytvela given in the current study (3 vs. 2 mg/kg) and the potentially faster route of drug uptake (intraperitoneal vs. subcutaneous), the rytvela treatment used exhibited limited efficacy in reducing transcriptional cytokine gene changes; this should be considered in the context of varying kinetics and mechanisms of the inflammatory response between species and treatment regimens. In future studies, extending the treatment window may be advantageous.

## 5 Conclusion

Pregnant spiny mice administered rytvela showed a partial reduction in increased maternal IL‐1β concentrations following subclinical inflammation induced by the viral mimetic Poly(I:C) in mid‐gestation. While rytvela was associated with improved postnatal growth following in utero Poly(I:C) challenge, a decrease in offspring gross locomotor activity after rytvela exposure warrants further investigation.

## Author Contributions

Nhi T. Tran: data acquisition, data analysis, data visualization, initial drafting of manuscript, editing and approval of the final draft; Rachid Kamareddine: data acquisition, data analysis, editing and approval of the final draft; Nadia Bellofiore: data acquisition, data analysis, editing and approval of the final draft; David M. Olson: conception and design of the study, funding acquisition, editing and approval of the final draft; Sylvain Chemtob: conception and design of the study, editing and approval of the final draft; Sarah A. Robertson: conception and design of the study, funding acquisition, editing and approval of the final draft; Jeffrey A. Keelan: conception and design of the study, funding acquisition, editing and approval of the final draft; Stacey J. Ellery: conception and design of the study, data acquisition, data analysis, initial drafting of manuscript, funding acquisition, editing and approval of the final draft

## Funding

This study was supported by an Australian NHMRC Project Grant (1145295).

## Conflicts of Interest

The authors declare no conflicts of interest.

## Supporting information




**Supplementary Tables**: dneu70064‐sup‐0001‐TableS1‐S4.docx

## Data Availability

All data are available in the main text or the supplementary materials. Data from this study are available from the corresponding author upon reasonable request.
