# Supplementary material for: Maternal and Postnatal Effects of Poly I:C‐Induced Intrauterine Inflammation and Rytvela, a Non‐Competitive IL‐1R Antagonist, in the Pregnant Spiny Mouse
Source: Dev Neurobiol. 2026 Sep 17;86(4):e70064. doi: 10.1002/dneu.70064 (PMC13586770; doi:10.1002/dneu.70064)
Supplement: Supplementary file 1 [file DNEU-86-0-s001.docx]

Supplementary Materials

**Table S1. RT-qPCR primers**

| **Gene** | **Forward primer sequence (5′–3′)** | **Reverse primer sequence (3′–5′)** |
| --- | --- | --- |
| *IL1B* | CACCTTCTTTTCCTTCATCTTTG | CACACCAGCAGGTTATATCA |
| *IL6* | CAGAGCCATTCAGAGCAACA | TGACCTGGATCCTTCACCTC |
| *NFKB1* | TGAGGGATCTGCTGGAAGTC | CCAAGTGCAGAGGTGTCTGA |
| *TNF* | CAAATTCGAGTGACAAGCCTG | GAGATCCATCCCGTTGGC |
| *Rn18s1* | ACACGGACAGGATTGACAGA | CAAATCGCTCCACCACCAACTAA |

**Table S2: Validation steps for ELISA detection of IL-1β in spiny mouse amniotic fluid**

| **SPIKE AND RECOVERY: AMNIOTIC FLUID** | | | | | **LINEARITY OF DILUTION: AMNIOTIC FLUID**  **Expected: 1000 pg/mL** | | | | |
| --- | --- | --- | --- | --- | --- | --- | --- | --- | --- |
|  | |  |  |  |  |  |  |  |  |
|  | ***HIGH (Expected: 1000 pg/mL)*** | ***LOW (Expected 4.12 pg/mL)*** | | |  |  |  |  |  |
|  | *Recovery (%)* | *Recovery (%)* | | | *Concentration* | *Recovery (%)* |  |  |  |
| Sample 1 | 94.6 | 128.7 | | | Neat | 102.4 | | | |
| Sample 2 | 97.7 | 153.6 | | | 1/2 | 129.4 | | | |
| Sample 3 | 114.8 | 139.2 | | | 1/4 | 133.3 | | | |
| *Average* | *102.4* | *140.4* | | |  |  |  |  |  |
| *Amniotic Fluid Average* | *102.4+140.4/2 =* ***121.5%*** |  | | | Dilution Used | **Neat** |  |  |  |

**Table S3:** **Validation steps for ELISA detection of IL-1β in spiny mouse serum**

| **SPIKE AND RECOVERY: AMNIOTIC FLUID** | | | | | **LINEARITY OF DILUTION: AMNIOTIC FLUID** | | | | |
| --- | --- | --- | --- | --- | --- | --- | --- | --- | --- |
|  | |  |  |  |  |  |  |  |  |
|  | ***HIGH*** | ***LOW*** | | |  |  |  |  |  |
|  | *Recovery (%)* | *Recovery (%)* | | | *Concentration* | *Recovery (%)* |  |  |  |
| Sample 1 | 141.4 | 111.7 | | | Neat | 109.0 | | | |
| Sample 2 | 88.9 | 126.8 | | | 1/2 | 106.3 | | | |
| Sample 3 | 123.1 |  | | | 1/4 | 130.4 | | | |
| *Average* | *117.8* | *119.2* | | |  |  |  |  |  |
| *Serum Average* | *117.8.+119.2/2 =* ***118.5%*** |  | | | Dilution Used | **1/2** |  |  |  |

**Table S4. Body and organ weights of spiny mice offspring at PND45**.

Data are presented as mean ± SD. Data were analysed by one-way ANOVA for parametric data and a Kruskal-Wallis test for non-parametric data. *p*-value<0.05 considered significant. BW=body weight

|  | Control  (*n*=7) | Poly(I:C)  (*n*=8) | Poly(I:C) + Rytvela  (*n*=11) | *ANOVA or* *Kruskal-Wallis P value* |
| --- | --- | --- | --- | --- |
| Body (g) | 30.58 ± 2.28 | 30.86 ± 3.96 | 31.33 ± 1.87 | *F*(2, 23)=1.518, *P*=0.85 |
| Brain (% BW) | 2.52 ± 0.15 | 2.52 ± 0.30 | 2.41 ± 0.18 | *H*(2)=1.846, *P*=0.41 |
| Liver (% BW) | 3.41 ± 0.19 | 3.90 ± 0.59 | 3.78 ± 0.72 | *H*(2)=5.068, *P*=0.08 |
| Thymus (% BW) | 0.13 ± 0.07 | 0.16 ± 0.04 | 0.14 ± 0.04 | *F*(2, 23)=0.499, *P*=0.64 |
| Spleen (% BW) | 0.21 ± 0.05 | 0.40 ± 0.05 | 0.20 ± 0.07 | *H*(2)=2.446, *P*=0.29 |
